# Can kinematic parameters of 3D reach-to-target movements be used as a proxy for clinical outcome measures in chronic stroke rehabilitation? An exploratory study

**DOI:** 10.1186/s12984-020-00730-1

**Published:** 2020-08-08

**Authors:** Catherine Adans-Dester, Susan E. Fasoli, Eric Fabara, Nicolas Menard, Annie B. Fox, Giacomo Severini, Paolo Bonato

**Affiliations:** 1 Department of Physical Medicine & Rehabilitation, Harvard Medical School, Spaulding Rehabilitation Hospital, 300 First Ave, Charlestown, Boston, MA 02129 USA; 2 School of Health & Rehabilitation Sciences, MGH Institute of Health Professions, Boston, MA USA; 3 Wyss Institute for Biologically Inspired Engineering, Harvard University, Boston, MA USA; 4 School of Electrical and Electronic Engineering, University College Dublin, Dublin, Ireland; 5 Centre for Biomedical Engineering, University College Dublin, Dublin, Ireland

**Keywords:** Clinical outcomes, Kinematics, Rehabilitation, Robot-assisted therapy, Reach-to-target, Stroke, Upper extremity

## Abstract

**Background:**

Despite numerous trials investigating robot-assisted therapy (RT) effects on upper-extremity (UE) function after stroke, few have explored the relationship between three-dimensional (3D) reach-to-target kinematics and clinical outcomes. The objectives of this study were to 1) investigate the correlation between kinematic parameters of 3D reach-to-target movements and UE clinical outcome measures, and 2) examine the degree to which differences in kinematic parameters across individuals can account for differences in clinical outcomes in response to RT.

**Methods:**

Ten chronic stroke survivors participated in a pilot RT intervention (eighteen 1-h sessions) integrating cognitive skills training and a home-action program. Clinical outcome measures and kinematic parameters of 3D reach-to-target movements were collected pre- and post-intervention. The correlation between clinical outcomes and kinematic parameters was investigated both cross-sectionally and longitudinally (i.e., changes in response to the intervention). Changes in clinical outcomes and kinematic parameters were tested for significance in both group and subject-by-subject analyses*.* Potential associations between individual differences in kinematic parameters and differences in clinical outcomes were examined.

**Results:**

Moderate-to-strong correlation was found between clinical measures and specific kinematic parameters when examined cross-sectionally. Weaker correlation coefficients were found longitudinally. Group analyses revealed significant changes in clinical outcome measures in response to the intervention; no significant group changes were observed in kinematic parameters. Subject-by-subject analyses revealed changes with moderate-to-large effect size in the kinematics of 3D reach-to-target movements pre- vs. post-intervention. Changes in clinical outcomes and kinematic parameters varied widely across participants.

**Conclusions:**

Large variability was observed across subjects in response to the intervention. The correlation between changes in kinematic parameters and clinical outcomes in response to the intervention was variable and not strong across parameters, suggesting no consistent change in UE motor strategies across participants. These results highlight the need to investigate the response to interventions at the individual level. This would enable the identification of clusters of individuals with common patterns of change in response to an intervention, providing an opportunity to use cluster-specific kinematic parameters as a proxy of clinical outcomes.

**Trial registration:**

ClinicalTrials.gov, NCT02747433. Registered on April 21st, 2016

## Introduction

Every year, about 795,000 people suffer a new or recurrent stroke in the United States [1] leading to hemiparesis and significant effects on the functional use of the paretic arm and hand [2]. Despite treatment, upper-extremity (UE) motor impairments and limited abilities to reach for and manipulate objects persist [3]. Less than half of the individuals who experience a stroke and severe UE hemiparesis in the acute phase regain purposeful UE function after 6 months [4, 5].

A large body of literature based on motor learning theories has shown that high-intensity, high-dosage rehabilitation interventions can facilitate sensorimotor recovery in stroke survivors [6–9]. Guidelines [10] recommend that the response to rehabilitation interventions be assessed across domains of the International Classification of Functioning, Disability and Health (ICF) [11]. Accordingly, clinical research studies report the results of rehabilitation interventions via a collection of standardized clinical outcome measures of UE function (e.g., Fugl-Meyer Assessment Upper Extremity subscale [FMA-UE] [12], Wolf Motor Function Test [WMFT] and Functional Ability Scale [WMFT-FAS] [13, 14]), and measures of UE activity performance in the home (e.g., Motor Activity Log [MAL] [15]).

Researchers and clinicians have investigated the use of kinematic parameters of UE movements as a proxy for clinical outcome measures after stroke [16–18]. This interest has been motivated by the development of rehabilitation technologies (e.g., robotic training devices and wearable sensing technologies) to collect data during the performance of UE movements and the need for precise and valid measures of UE motor function. While many researchers have used kinematic parameters to study two-dimensional (2D) UE reaching movements, few have studied the kinematics of three-dimensional (3D) movements [19–21].

Kinematic parameters derived via tracking of 2D arm reaching movements moderately correlated with FMA-UE scores, WMFT and WMFT-FAS scores, and self-reports of the amount of use (MAL-AOU) and quality of movement (MAL-QOM) of the hemiparetic limb [19–21]. Moderate correlation was also shown between clinical scores and kinematic parameters of UE movements performed using a rehabilitation robot. Seminal work by Rohrer et al. [22] showed that improvements in robot-based kinematic parameters aimed to capture the smoothness of arm reaching movements moderately correlated with changes in FMA-UE scores in response to robot-assisted intervention. Colombo et al. [23–25] further demonstrated a moderate correlation between robot-based kinematic parameters and FMA-UE scores; work by Zollo et al. [26], Otaka et al. [27], Duret et al. [28, 29], and Pila et al. [30] made comparable observations. Other authors achieved similar results by collecting kinematic data during the performance of tasks consisting of drawing geometric figures of different shapes [31, 32], tracing large semicircular arcs to measure UE active range of motion [33], or deriving kinematic parameters from distal movements (e.g., wrist and finger movements) [34]. Larger correlations were shown by Krebs et al. [35] when implementing more complex analytical models than those utilized in previous work. Interestingly, despite the moderate correlation coefficients between clinical scores and kinematic parameters at the group level, studies reporting data on a subject-by-subject basis showed a significant variability across individuals [22, 23, 28].

Because they are more relevant from a functional point of view than 2D movements, several authors have focused their efforts on the analysis of 3D movements and the associations between their kinematic parameters and clinical outcome measures. Seminal work by Cirstea [36] showed a strong correlation between joint kinematics of 3D arm-reaching movements and FMA-UE scores. However, these results are in conflict with later studies showing moderate-to-poor associations between 3D arm-reaching kinematics and FMA-UE scores [37–39]. Other research of UE kinematics during the performance of simulated drinking from a glass [40–44] also reported moderate-to-poor correlations with clinical outcome measures of motor impairment and activity performance. Subject-by-subject kinematic analyses are not typically reported for 3D arm-reaching movements and are needed to examine individual differences that contribute to these low associations. Specifically, individual analyses may reveal the degree to which changes in UE activity performance may be attributed to the restitution of motor function or use of compensatory movement strategies after stroke. Additionally, little is known about the association between changes in the kinematic parameters and changes in the clinical outcome measures observed in response to the intervention.

To that end, we analyzed pilot data collected during 3D reach-to-target movements in chronic stroke survivors who received a novel robot-assisted therapy protocol to 1) investigate the correlation between kinematic parameters of three-dimensional (3D) reach-to-target movements and UE clinical outcome measures, both cross-sectionally and longitudinally, and 2) examine the degree to which differences in kinematic parameters across individuals can account for differences in clinical outcomes in response to RT.

## Methods

The study was approved by Partners Healthcare Institutional Review Board and registered on ClinicalTrials.gov (NCT02747433). All participants provided written informed consent according to the Declaration of Helsinki.

### Participants

Ten chronic stroke survivors participated in the study [45]. The study inclusion criteria were: 1) chronic unilateral stroke (> 6 months); 2) moderate UE hemiparesis (baseline FMA-UE score between 21 and 50 out of 66 points) [12]; and 3) cognitive function adequate to understand and actively engage in the research procedures (Montreal Cognitive Assessment Score ≥ 26) [46]. The exclusion criteria were: 1) more than moderate impairments in paretic UE sensation, passive range of motion, and pain; 2) considerable muscle tone (Modified Ashworth Scale ≥3) [47]; 3) hemispatial neglect or visual field loss; 4) aphasia limiting comprehension and completion of the treatment protocol; 5) concomitant UE therapy; and 6) contraindications to RT. Table 1 shows the baseline characteristics of the study participants.

**Table 1:** Baseline characteristics of the study participants

Subject	Age (years)	Gender	Time since CVA (months)	Hemiparetic Side	Hand Dominance
1	25	Male	8.6	Right	Right
2	61	Male	10.9	Left	Right
3	31	Female	59.5	Right	Left
4	59	Male	188.8	Left	Left
5	81	Male	8.3	Right	Right
6	50	Male	7.1	Left	Left
7	64	Female	172.9	Right	Right
8	24	Female	99.7	Left	Right
10	73	Male	13.8	Left	Right
11	57	Female	17.9	Right	Right

### Intervention

Study volunteers participated in the Active Learning Program for Stroke (ALPS) in which one-on-one sessions with an occupational therapist consisted of cognitive strategy training and individualized home programs aimed to facilitate the transfer of robot-trained UE movements to functional use of the paretic arm and hand during everyday tasks. In this development of concept study, the repetitive movement therapy was delivered using two commercially available devices: the ArmeoSpring® (Hocoma AG, Volketswil, Switzerland) and the Amadeo® (Tyromotion, Graz, Austria). All participants received 18 one-hour sessions of therapy over a period of 6–7 weeks. Additional details concerning the intervention are reported elsewhere [45].

### Clinical outcome measures

Subjects were assessed at baseline and at the end of the intervention using clinical outcome measures across domains of the International Classification of Functioning, Disability and Health (ICF) [11]. A single rater, blinded to the group assignment, performed all assessments. UE motor function was assessed via observation using the Fugl-Meyer UE Assessment (FMA-UE) [12]. The Wolf Motor Function Test (WMFT) was used to assess activity limitations of the paretic arm and the quality of movement, as rated using the Functional Ability Scale (WMFT-FAS) [13, 14] . The Motor Activity Log was used to collect information about self-perceived amount of use (MAL-AOU) and quality of movement (MAL-QOM) of the paretic UE during daily activities [15].

### Kinematics of reach-to-target movements

#### Experimental protocol

**Fig. 1:**
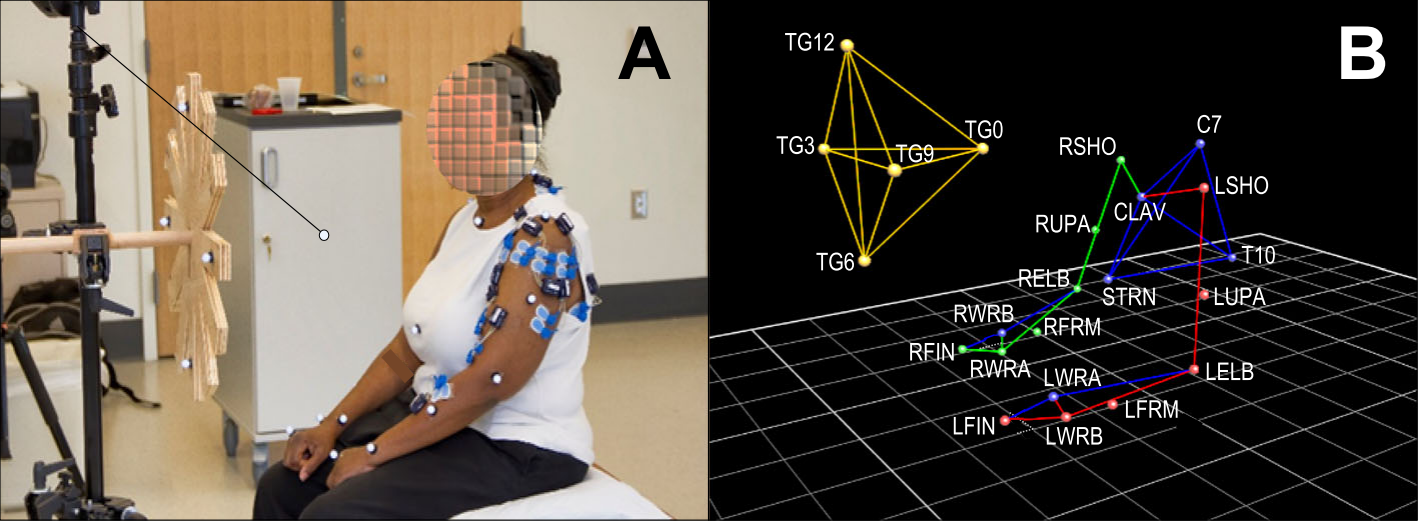
Experimental Set-up. **a***. Subject set-up:* Twenty reflective markers were placed on the following body landmarks: 7th cervical vertebra spinous process, 10th thoracic vertebra spinous process, suprasternal notch and xiphoid process. Markers were also placed bilaterally on the acromion, upper-arm, lateral epicondyle of the humerus, forearm, radial styloid process, ulnar styloid process, first metacarpal head and second metacarpal head. **b**. Biomechanical model of subject in 1**a** and the target panel: Biomechanical model (Plug-in-gait) applied to reconstruct UE segments and derive kinematic parameters

A 10-camera motion capture system (Vicon, Oxford Metrics Ltd., Oxford, UK) was used to track reflective markers placed on the upper body during reach-to-target movements (Fig. 1). The data was recorded at a rate of 120 frames per second.

The subjects were seated on a bench without back support so as not to constrain movement and faced a vertical panel (Fig. 1a). The panel had twelve numbered targets positioned in a clock-like fashion 20 cm from its center, which was aligned with the acromion of the arm being tested. To determine the position of the targets in the 3D calibrated volume of the motion capture system, four reflective markers were placed on the back of the panel. The distance between the subject and the center of the panel was set according to each subject’s arm length measured with the fist closed. To standardize the starting position of the reaching-to-target movements, an additional target was positioned at forearm distance (measured from the lateral epicondyle to the ulnar styloid process) from the center of the panel along the virtual line connecting the center of the panel and the shoulder acromion.

During each trial, study participants were instructed to reach at a self-selected speed to specific numbers on the panel. Each movement was performed with a closed fist from the starting position to the selected target and then back to the starting position. The target order was randomized, and the same sequence was used across sessions. Subjects were allowed to rest as needed. The data analyses described in the following were based on 36 reach-to-target movements performed with the paretic UE and 36 reach-to-target movements performed with non-paretic UE by each subject.

#### Data processing

The Nexus 2.8.1 software (Vicon, Oxford Metrics Ltd., Oxford, UK) was used to derive the movement kinematics from the position data of the UE reflective markers. The start and end of the reach-to-target movements were identified using the marker positioned on the metacarpal head of the index finger; events were manually verified. Joint angles were estimated using the Nexus 2.8.1 software via a standard biomechanical model in which anatomical joints are represented as universal joints.

The data was then imported to MATLAB (The MathWorks Inc., Natick MA, USA) and custom scripts were used to derive the eleven kinematic parameters listed in Table 2. These kinematic parameters were selected based on recent recommendations to enhance standardization of UE kinematic studies in stroke survivors [16].

**Table 2:** Kinematic Constructs and Parameters Extracted

Construct	Parameter	Unit	Definition
Efficiency	**Movement Time** (MT)	Seconds	Time elapsed between movement onset and end^a^
Accuracy	**Trajectory Directness** (CurvI)		Ratio of the actual movement trajectory between movement initiation and final position, and the straight line joining those two events^a^
Speed	**Peak Velocity** (V_max_)	cm/s	Maximum velocity^a^
Planning	**Time to Peak Velocity** (T%V_max_)	Percentage	Time to achieve maximum velocity, expressed as percentage of the movement duration^a^
Smoothness	**Number of Velocity Peaks** (NVP)		Number of peaks of the movement velocity trajectory
	**Log Dimensionless Jerk** (LDJ)		Negative logarithm of the dimensionless jerk metric
Joint Range of Motion	**Range Shoulder Flexion/Extension** (ShFE)	Degrees	Range of the shoulder flexion/extension angle between movement onset and end
	**Range Shoulder Abduction/Adduction** (ShAA)	Degrees	Range of the shoulder abduction/adduction angle between movement onset and end
	**Range Elbow** **Flexion/Extension** (ElFE)	Degrees	Range of the elbow flexion/extension angle between movement onset and end
Trunk Movement	**Range Thorax** (Th)	Degrees	Angle of rotation of the shoulders in respect to a projected line between the shoulder markers
	**Torso Excursion** (TExc)	cm	Displacement of the trunk, measured with the clavicle marker

^a^Variable calculated using the marker positioned on the metacarpal head of the index finger

The marker positioned on the metacarpal head of the index finger was also used to derive the following six parameters. The trajectory directness [48] and the movement time duration were computed to capture movement accuracy and efficiency. The time to peak velocity and the peak velocity provided information on movement planning and speed, respectively. Movement smoothness was quantified by computing the total number of velocity peaks [48] and the log dimensionless jerk [49] of the marker trajectory. Finally, a subset of markers positioned on the UE and trunk was used to estimate shoulder and elbow range of motion. Compensatory trunk movements were captured by estimating the displacement of the trunk in the transverse and sagittal plane.

### Statistical analysis

Spearman rank tests were used to assess correlations between the kinematic variables and clinical outcome measures. These analyses were performed both cross-sectionally and longitudinally. Cross-sectional analyses (i.e. pre- and post-intervention assessments) examined whether kinematic parameters were correlated with the severity of motor impairments and functional limitations captured by the clinical outcome measures. Longitudinal analyses (i.e. pre- vs. post-intervention assessments) were performed to determine if the changes in the kinematic parameters and clinical outcome measures following the intervention were correlated. These correlation tests were carried out both for total clinical scores (i.e., using all items of the scales) and for scores associated with the assessment of proximal body segments (i.e., using only items to assess shoulder and elbow), as the evaluated kinematics did not measure specific movements of distal body segments (e.g., forearm pro/supination, wrist and hand motions). Correlation coefficients were labeled as high (0.70 to 1.00), moderate (0.50 to 0.70), low (0.30 to 0.50) or negligible (0.00 to 0.30), according to the “rule of thumb” proposed by Hinkle et al. [50].

Paired t-tests were performed at the group level to determine if the clinical outcome measures and kinematic parameters collected during the study significantly changed in response to the intervention. Because of the small sample size, we used bootstrapping with 1000 samples and percentile-corrected confidence intervals for these analyses.

In addition, subject-by-subject analyses were performed for both the kinematic parameters and clinical outcome measures to examine changes occurring at the individual level using paired t-tests with bootstrapping. Cohen’s *d* effect size values were calculated for each comparison using the pre-intervention standard deviation as the denominator in the calculation of Cohen’s *d*. We used Cohen’s conventions of *d* = 0.20, 0.50, and 0.80, representing small, medium, and large effect sizes, respectively [51]. Clinical outcome measures were also examined at the individual level. Minimal clinically important difference (MCID) values were identified from the literature [52–54].

Statistical tests were performed using SPSS (Statistical Packages for Social Sciences, version 26.0; SPSS Inc., Chicago, IL, USA). Significance was set a priori at α = 0.05 and *p*-values were adjusted for multiple comparisons using a Holm correction [55]. In the interpretation of the results, the emphasis will be on effect sizes due to the exploratory nature of the study and the small sample size.

## Results

### Correlation analyses

Both cross-sectional and longitudinal correlation analyses examined associations between kinematic parameters and clinical outcome scores. Table 3 summarizes the results of these analyses.

**Table 3:** Correlation analyses between clinical outcomes and kinematic variables (Spearman *r*_*s*_), panel **A** reports cross-sectional analyses and panel **B** longitudinal analyses

***A.***		**MT**	**Curvl**	**V**_**max**_	**T%V**_**max**_	**NVP**	**LDJ**	**ShFE**	**ShAA**	**ElFE**	**Th**	**TExc**
**Total**	FMA-UE	−0.50^⁑^	− 0.32^⁑^	0.22^*^	0.11	−0.30^⁑^	0.34^⁑^	0.20^*^	−0.14	*0.64*^⁑^	**−0.73**^⁑^	*−0.58*^⁑^
	WMFT-FAS	*−0.51*^⁑^	−0.34^⁑^	0.12	0.06	−0.39^⁑^	0.41^⁑^	0.22^*^	−0.11	*0.64*^⁑^	*−0.63*^⁑^	*−0.57*^⁑^
	WMFT-Time	0.45^⁑^	0. 26^⁑^	0.01	−0.13	0.32^⁑^	−0.36^⁑^	−0.11	0.15	−0.43^⁑^	*0.53*^⁑^	0.37^⁑^
**Proximal**	FMA-UE	−0.47^⁑^	−0.36^⁑^	0.19^*^	0.08	−0.32^⁑^	0.39^⁑^	0.22^⁑^	−0.14	**0.74**^⁑^	**−0.78**^⁑^	**−0.72**^⁑^
	WMFT-FAS	*−0.57*^⁑^	−0.38^⁑^	0.20^*^	−0.02	− 0.45^⁑^	0.47^⁑^	0.24^⁑^	−0.11	*0.68*^⁑^	*−0.67*^⁑^	*− 0.61*^⁑^
	WMFT-Time	*0.58*^⁑^	0.39^⁑^	−0.27^⁑^	0.07	*0.51*^⁑^	*−0.55*^⁑^	−0.11	0.09	*−0.64*^⁑^	*0.58*^⁑^	*0.60*^⁑^
***B.***		Δ **MT**	Δ **Curvl**	Δ **V**_**max**_	Δ **T%V**_**max**_	Δ **NVP**	Δ **LDJ**	Δ **ShFE**	Δ **ShAA**	Δ **ElFE**	Δ **Th**	Δ **TExc**
**Total**	Δ FMA-UE	−0.46^⁑^	−0.15	−0.07	0.20	−0.42^⁑^	0.48^⁑^	*−0.61*^⁑^	−0.27^*^	− 0.16	−0.07	− 0. 37^⁑^
	Δ WMFT-FAS	−0.03	0.02	0.05	−0.41^⁑^	−0.20	0.16	−0.12	− 0.04	−0.20	0.22	0.29^*^
	Δ WMFT-Time	0.39^*^	−0.01	0.14	−0.44^⁑^	0.24	−0.20	0.46^⁑^	0.45^⁑^	0.07	−0.07	0.43^⁑^
**Proximal**	Δ FMA-UE	−0.37^⁑^	−0.09	− 0.04	0.13	− 0.33^⁑^	0.36^⁑^	*− 0.63*^⁑^	−0.28^*^	− 0.18	−0.00	− 0.23
	Δ WMFT-FAS	−0.05	0.09	0.17	−0.41^⁑^	−0.23	0.18	−0.12	− 0.03	−0.22	0.20	0.30^*^
	Δ WMFT-Time	−0.07	−0.12	0.25	−0.11	0.04	0.10	0.26	0.30^*^	−0.16	−0.24	− 0.03

* test significant at *p* < 0.05, ^⁑^ at *p* < 0.01 after Holm-adjustment. **Bold** when high correlations (*r*_*s*_ > 0.70); *italic* when moderate (0.70 > *r*_*s*_ > 0.50); *r*_*s*_ values lower than 0.50 are low

Δ: changes (post-pre); MT: movement time (s); Curvl: trajectory directness; V_max_: peak velocity (cm/s); T%V_max_: peak velocity (%); NVP: number of velocity peaks; LDJ: log dimensionless jerk; ShFE: range shoulder flexion/extension (deg); ShAA: range shoulder abduction/adduction (deg); ElFE: range elbow flexion/extension (deg); Th: range of thorax rotation (deg); TExc: maximum distance travelled by the clavicle marker (cm)

The upper panel (A) of Table 3 shows the results of cross-sectional correlation tests. Low to moderate correlation coefficients (*r*_*s*_ < 0.70) were observed for the majority of the comparisons. Although statistically significant associations were identified between many kinematic parameters and clinical outcome measures, high correlation coefficients (*r*_*s*_ > 0.70) were only found between elbow flexion-extension, thorax rotation, and torso excursion and a clinical measure of UE impairment (FMA-UE).

The lower panel (B) of Table 3 shows the results of the correlation analyses for the longitudinal data (i.e. correlation between changes in kinematic parameters and changes in clinical outcome measures in response to the intervention). Low and non-significant correlation coefficients (*r*_*s*_ < 0.50) were found between the changes in the kinematic parameters and clinical outcome measures following the intervention, except for a negative moderate association between the FMA-UE and shoulder flexion-extension.

### Group analyses

Paired t-tests revealed significantly improved motor function with a large and medium-to-large effect size for all clinical outcome measures, except for the performance time (WMFT-Time) which displayed a small effect size (Table 4). Detailed analyses of the clinical outcomes have been reported elsewhere [45].

**Table 4:** Clinical outcome measures pre- vs. post-intervention (group analyses - *N* = 10)

Outcome	Pre-intervention		Post-intervention		Cohen’s ***d***
	Median [Q_**1**_; Q_**3**_]	Mean ± SD	Median [Q_**1**_; Q_**3**_]	Mean ± SD	
FMA-UE^a^ (points)	32.00 [22.50; 41.50]	32.20 ± 9.60	42.50 [31.75; 48.50]	39.50 ± 10.01	0.76*
WMFT-Time^b^ (s)	18.03 [14.81; 56.00]	32.82 ± 25.33	14.18 [8.08; 35.30]	24.04 ± 23.54	0.35*
WMFT-FAS^c^ (points)	2.77 [1.90; 2.82]	2.39 ± 0.64	3.17 [1.97; 3.51]	2.83 ± 0.77	0.71*
MAL-AOU^d^ (points)	1.01 [0.52; 1.93]	1.19 ± 0.73	1.9 [1.32; 2.61]	2.01 ± 0.86	1.12*
MAL-QOM^e^ (points)	0.99 [0.62; 1.93]	1.21 ± 0.64	1.99 [1.42; 2.65]	2.05 ± 0.78	1.31*

^a^Points out of 66. Higher scores indicate reduction of impairments;

^b^Average time required to perform the tasks in seconds. Lower scores indicate improved task completion

^c^Points out of 5. Higher scores indicate improvement in quality of movement

^d^Points out of 5. Higher scores indicate improved perceived usage of UE

^e^Points out of 5. Higher scores indicate improved perceived quality of UE use

* Bootstrapped test significant (*p* < 0.01) after Holm-adjustment

Cohen’s *d* effect size: *d* > 0.30 (small), *d* > 0.50 (medium) and *d >* 0.80 (large)

Paired t-test analyses did not show any significant changes in kinematic parameters pre- to post-intervention. Also, the effect size for most of the kinematic parameters was small. Only the time to peak velocity (*T%V*_*max*_) was marked by a medium-to-large effect size and the change in log dimensionless jerk (*LDJ*) was marked by a small-to-medium effect (Table 5). These findings suggest that, at the group level, subjects did not change consistently their movement strategies (as measured by kinematics) during 3D reach-to-target movements in response to the intervention.

**Table 5:** Kinematic parameters pre- and post-intervention (group analyses – *N* = 10)

Construct	Kinematic parameter	Pre-intervention		Post-intervention		Cohen’s ***d***
		Median [Q_**1**_; Q_**3**_]	Mean ± SD	Median [Q_**1**_; Q_**3**_]	Mean ± SD	
Efficiency	MT *(s)*	2.37 [1.95; 2.86]	2.41 ± 0.58	2.17 [1.94 ;2.51]	2.28 ± 0.69	0.24
Accuracy	CurvI	1.43 [1.34; 1.64]	1.67 ± 0.69	1.54 [1.36; 1.73]	1.88 ± 1.14	0.30
Speed	V_max_*(cm/s)*	50.19 [44.53; 61.63]	55.72 ± 17.08	54.07 [45.95; 73.32]	58.44 ± 16.18	0.16
Planning	T%V_max_*(%)*	41.98 [26.98; 50.95]	39.11 ± 12.21	48.85 [42.88; 52.56]	47.67 ± 6.13	0.70
Smoothness	NVP	15.41 [13.07; 23.73]	17.11 ± 5.27	13.45 [10.71; 21.63]	15.55 ± 5.66	0.29
	LDJ	−18.89 [−19.66; -18.25]	−18.96 ± 0.80	−18.14 [−19.21; -17.97]	− 18.60 ± 1.38	0.46
Joint Range of Motion	ShFE *(deg)*	19.67 [14.91; 22.40]	18.86 ± 4.67	20.29 [12.09; 24.71]	18.61 ± 7.32	0.05
	ShAA *(deg)*	25.58 [20.39; 28.39]	25.14 ± 4.82	27.12 [18.36; 29.53]	25.90 ± 8.37	0.16
	ElFE *(deg)*	39.64 [16.61; 60.80]	38.89 ± 22.73	39.24 [17.99; 46.60]	36.84 ± 19.49	0.09
Trunk Movement	Th *(deg)*	5.46 [3.22; 9.85]	6.61 ± 4.07	5.40 [3.09; 9.92]	6.97 ± 5.09	0.09
	TExc *(cm)*	11.02 [6.77; 16.19]	12.83 ± 7.91	9.53 [6.32; 17.32]	12.56 ± 8.68	0.03

Cohen’s *d* effect size: *d* > 0.30 (small), *d* > 0.50 (medium) and *d* > 0.80 (large). No bootstrapped tests were significant (*p* < 0.05) after Holm-adjustment.

### Subject-by-subject analyses

In attempt to investigate why changes in response to the intervention were clearly identified in the analysis of the clinical outcome measures whereas no consistent changes were shown by the kinematic parameters of UE movements, clinical outcome measures and kinematic parameters were examined and compared on a subject-by-subject basis.

**Fig. 2:**
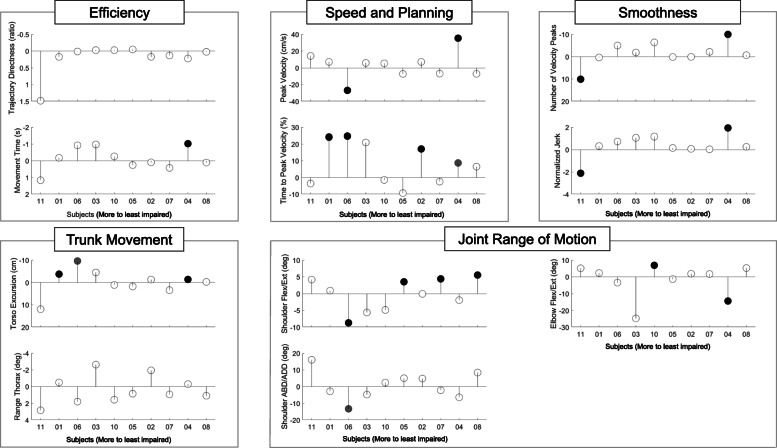
Changes in kinematic parameters pre- vs. post-intervention (subject-by-subject analyses). Improvements are represented above the horizontal line. Circles filled in grey and black represent statistically significant changes after Holm-Adjustment at *p*<0.05 and *p*<0.005, respectively. Unfilled circles represent changes that did not reach statistical significance.

Figure 2 provides a graphical representation of results of the paired t-tests performed at the individual level, using data collected for all targets pre- vs. post-intervention. Statistically significant improvements were observed most frequently across study participants for the time to peak velocity (*T%V*_*max*_) and torso excursion (*TExc*) as seen in Fig 2 (speed and planning and trunk movement panels, respectively). The large effect size estimates associated with these parameters shown in Table 6 suggest that the intervention was effective in facilitating the planning and execution of reach-to-target movements and improving the isolation of trunk and UE motions during reach on an individual basis. The other kinematic parameters significantly changed following intervention in less than half of the participants. Figure 2 and Table 6 illustrate the high degree of variability among participants. This high variability likely contributed to the small kinematic changes evidenced at the group level.

**Table 6:**
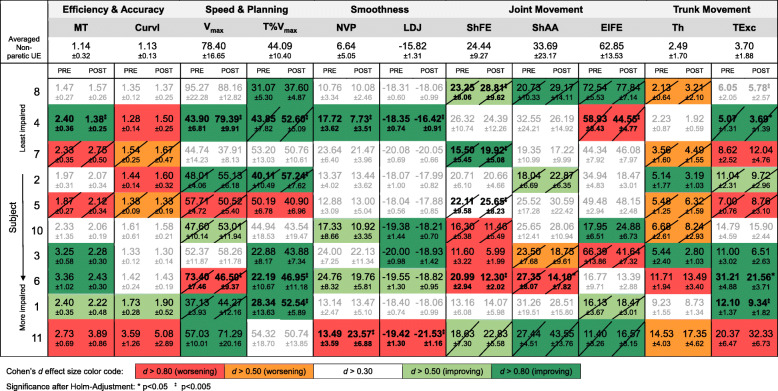
Kinematic parameters pre- vs. post-intervention (subject-by-subject analyses)

At the individual level, all subjects displayed changes in kinematic parameters, some of which exhibited medium to large effect size. The first row of data in Table 6 provides group kinematic data for the non-paretic UE, to be used as a reference. When examining the data on a subject-by-subject basis (i.e., the individual rows of Table 6), it is apparent that the mean values (pre- and post-intervention) of several kinematic parameters for the paretic UE fell within the interval defined by the mean ± one standard deviation of the same kinematic parameter for the non-paretic UE. When changes pre- vs post-intervention in the kinematic parameter values for the paretic UE fell within such interval, they were not considered as noteworthy because of their similarity with the values measured during non-paretic reach-to-target movements. Furthermore, some of the statistically significant kinematic changes reported in Table 6 are small in magnitude and hence unlikely to be clinically important. We highlighted these cases in Table 6 (hatched cells of the table).

Examination of subject-by-subject changes in clinical outcome measures collected during the study also showed remarkably different responses to the intervention across individuals. Table 7 provides a summary of these data. It is worth noting that 9 out of 10 participants demonstrated clinically meaningful gains in response to the intervention, as indicated by the MCIDs for these outcome measures, and represented across ICF domains.

**Table 7:**
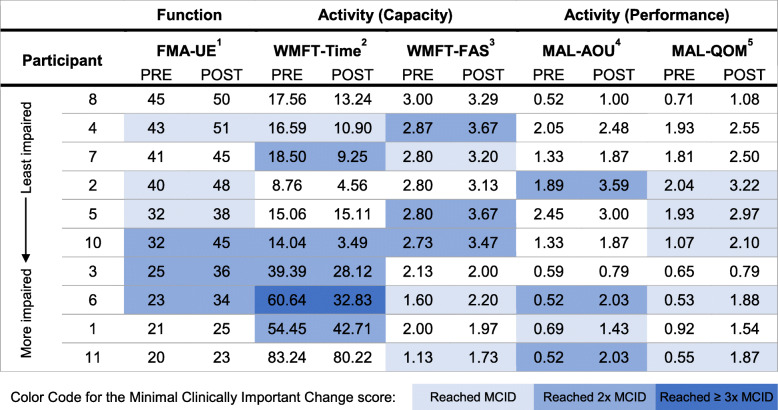
Clinical outcomes pre- vs. post-intervention (subject-by-subject analyses)

1 Points out of 66. Higher scores indicate reduction of impairments;

2 Average time required to perform the tasks. Lower scores indicate improved task completion.

3 Points out of 5. Higher scores indicate improvement in quality of movement.

4 Points out of 5. Higher scores indicate improved perceived usage of UE.

5 Points out of 5. Higher scores indicate improved perceived quality of UE use

## Discussion

This pilot study examined changes in kinematic parameters of 3D reach-to-target movements in response to an intervention that combined cognitive strategy training and individualized home programs to facilitate the transfer of robot-trained UE movements to functional use of the paretic arm and hand during everyday tasks. Two fundamental questions were addressed via group and subject-by-subject analyses: 1) What are the associations between the kinematic parameters of 3D reach-to-target movements and UE clinical outcome measures? and 2) Can differences in kinematic parameters across individuals account for differences in clinical outcomes in response to RT?

The kinematic analysis methods utilized in this pilot study provide the methodology to explore group and individual differences in response to interventions and to identify kinematic parameters that may be used as a proxy for clinical outcome measures. These methods may be used in a future larger clinical trial aimed to further differentiate the key treatment elements (e.g. dose, cognitive strategy training, protocol adherence) and personal characteristics (e.g. time post stroke, impairment level) associated with the recovery of motor function after stroke. The low to moderate correlation between individual kinematic parameters of 3D movements and clinical scores observed in this pilot study may be attributed to the three factors discussed below.

### Variability in response to the intervention

High variability in the response to UE intervention among these chronic stroke survivors likely contributed to the lack of statistically significant correlation as well as unexpected associations between clinical outcome measures and kinematic parameters at the group level. For instance, Table 3 shows a negative correlation between changes in shoulder flexion/extension range of motion and changes in FMA-UE scores in response to the intervention. This is counteractive from a clinical point of view. However, Fig. 2 and Table 6 highlight the fact that half of the study participants displayed a decrease and half an increase in shoulder flexion/extension range of motion pre- vs. post-intervention. Furthermore, Tables 6 and 7 show that the three subjects who displayed the largest decrease in shoulder flexion/extension range of motion (i.e., subjects 3, 6 and 10) are the same subjects who displayed the largest improvements in FMA-UE scores. This suggests that the distinct response observed in subjects 3, 6 and 10 and the small sample size of the study may be largely responsible for the negative correlation between changes in shoulder flexion/extension range of motion and changes in FMA-UE scores reported in Table 3. Figure 2 and Table 6 show that this correlation does not apply to the entire group.

Furthermore, by examining Fig. 2 and Table 6, one can identify additional subgroups of study participants displaying other common kinematic behaviors. For instance, none of the subjects displaying a change in torso excursion marked by large effect size showed an improvement in shoulder flexion/extension range of motion. Vice versa, subjects displaying an improvement in shoulder flexion/extension range of motion did not appear to rely on improving the control of torso excursion as part of their response to the intervention. These observations suggest the possibility that kinematic analyses may enable the identification of different motor strategies in response to the intervention (e.g., some subjects relying on improving the control of torso excursion vs. other subjects increasing shoulder flexion/extension range of motion). The results also suggest that different motor strategies might be adopted by individuals with different baseline motor impairments.

Importantly, these results highlight the shortcomings of group analyses as the ones reported in Table 3 when the results are marked by high variability in the kinematic and clinical response to the intervention. The subject-by-subject analyses presented in this manuscript are a departure from previous studies focused on group analyses [36–44] that we carried out in order to address the above-discussed shortcomings. We highlighted distinct changes in movement characteristics across individuals in response to the intervention. It is conceivable that a larger study, aimed to investigate variability across individuals, would enable the identification of clusters of participants with similar patterns of kinematic parameters during UE reaching tasks and similar changes in response to the intervention. These clusters would display higher levels of association between specific kinematic parameters and clinical outcome measures.

### Changes in motor strategy may be limited by the intervention dosage

Although statistically and clinically significant improvements were apparent on clinical outcome measures after only 18 sessions of therapy (Tables 4 and 7), consistent changes in motor strategy were not evident in the individual analyses of kinematic data (Table 6). The lack of consistent relationships observed between these two types of measures for participants with chronic stroke impairments, even at the individual level, suggests that kinematics may be a better indicator of “true” motor recovery and may distinguish between motor restitution and the use of dormant capacities or compensatory strategies that contribute to changes in clinical outcome measures. In fact, much of the current literature in the chronic stroke population fails to report results identifying “true” motor restitution as distinct from the unmasking of latent capacity, as studies often rely solely on clinical outcome measures to assess intervention effectiveness.

Would interventions marked by a higher-dosage and delivered over a longer time period have resulted in greater changes in motor strategies reflected in the kinematic parameters of arm reaching movements? While high dosage UE intervention studies after stroke [56, 57] may provide greater opportunities to improve motor function, as evidenced by clinical measures of performance, these high-dose studies have yet to be evaluated with kinematic analyses of UE task performance. Distinct patterns of changes in both clinical measures of performance and kinematic parameters could reflect “true” changes in UE motor strategy following intervention. In contrast, modest improvements in clinical measures of performance that are not accompanied by distinct changes in kinematic parameters may be more likely related to the unmasking of capabilities that were quiescent at the beginning of the intervention, or to the use of compensatory strategies [57, 58]. In this context, it would be interesting to compare results in chronic stroke survivors with results in a more acute population, which has the potential to display motor gains of greater magnitude and possibly clearer changes in motor strategy.

### A single task or kinematic parameter carries limited information

The kinematic parameters reported in this study were derived from a single 3D reach-to-target motor task, whereas clinical outcome assessments typically measure the functional use of the paretic arm and hand during a variety of movement tasks. This may contribute to a mismatch between the changes in motor abilities indicated by clinical outcome assessments and those that are kinematically derived from a single motor task. For instance, the kinematics of reach-to-target movements primarily measure the control of proximal body segments. In contrast, changes in the clinical outcome measures employed in the study also reflect changes in the control of distal body segments (i.e. manipulation tasks), which were not investigated by our kinematic analysis. Recent recommendations highlight the need for utilizing a battery of five motor tasks to assess changes observed in movement kinematics during clinical trials with a focus on sensorimotor recovery in stroke survivors [58]. Additionally, recent studies showing that wearable sensor data collected during functional tasks can accurately estimate clinical scores suggest that kinematic data collected across a variety of motor tasks may better serve as a proxy for estimating and tracking clinical outcome measures in future trials [59–61].

### Limitations and future studies

The results of this pilot study provide motivation to further explore the mechanisms of recovery during rehabilitation interventions in chronic stroke survivors. The small number of participants did not allow us the use of more complex statistical analyses and did not enable the identification of clusters of individuals who responded consistently to clinical interventions. In larger studies, analyses could be carried out separately for each cluster or at the group level using more complex regression models to examine possible relationships among the variables, which may be or not be linear. These models could include factors that may contribute to the relationship between kinematic variables and clinical outcomes, such as age, handedness, stroke lesion and chronicity. Larger studies would also allow for the kinematic investigation of 3D reach-to-target movements on a target-by-target basis (see plots in Supplementary Materials), further contributing to our understanding of motor recovery. Although these plots do not suggest obvious trends across targets, one would expect the identification of different clusters of motor performance (which may be associated with motor phenotypes) based on kinematic characteristics for individual targets. For example, reaching for ipsilateral targets with the paretic arm requires the inhibition of pathological flexor synergies; reaching for contralateral targets is facilitated by extensor synergies; and reaching for targets around the midline requires the modulation of both flexor and extensor synergies.

Further investigation of UE kinematics across an array of motor tasks that better reflect important clinical changes in the control of movement may contribute to greater understanding of motor function recovery. The use of standard sets of tasks and kinematic parameters to test motor capacity across studies will allow for better comparison across studies and a means to aggregate kinematic data for meta-analytic reviews [16, 58]. The development of wearable sensors to quantitatively evaluate UE movements where they matter the most, in the home-setting, has the potential to better measure UE function and limitations during activities of daily living. This would provide a more comprehensive evaluation of UE performance after stroke and a better characterization of the mechanisms underlying changes observed in clinical outcome measures.

### Conclusions

The results of this development-of-concept study showed large variability in the response to rehabilitation in a small sample of individuals with chronic motor impairments after stroke. Although statistically significant correlations were identified between kinematic parameters and clinical outcomes, correlation coefficients were not high. The high variability across kinematic parameters suggests no consistent pattern of change in UE motor strategies across participants. It is unclear if interventions marked by larger motor gains would display a more consistent change in kinematic behaviors. Nonetheless, our results support the need to further investigate the impact of interventions at the individual level. This would enable the identification of clusters of individuals with common patterns of change in kinematic parameters in response to an intervention, providing an opportunity to use cluster-specific kinematic parameters as a proxy of clinical outcomes.

## Supplementary information

**Additional file 1: Figure S1A.** Variance observed across targets in the kinematic variables of efficiency in the non-paretic and paretic arm. **Figure S1B.** Variance observed across targets in the kinematic variables of speed and planning in the non-paretic and paretic arm. **Figure S1C.** Variance observed across targets in the kinematic variables of smoothness in the non-paretic and paretic arm. **Figure S1D.** Variance observed across targets in the kinematic variables of trunk posture in the non-paretic and paretic arm. **Figure S1E.** Variance observed across targets in the kinematic variables of arm posture in the non-paretic and paretic arm.

## Data Availability

The dataset used in this manuscript is available upon request to be submitted to the corresponding author. The authors intend to post the dataset on PhysioNet https://physionet.org/.

## Abbreviations

ALPS: Active Learning Program for Stroke
CurvI: Trajectory Directness
ElFE: Range of Elbow Flexion-Extension
FMA-UE: Fugl-Meyer Upper Extremity Assessment
ICF: International Classification of Functioning, Disability and Health
MAL: Motor Activity Log
MT: Movement Time
LDJ: Log Dimensionless Jerk
NVP: Number of Velocity Peaks
RT: Robotic-Assisted Therapy
ShAA: Range of Shoulder Abduction-Adduction
ShFE: Range of Shoulder Flexion-Extension
Th: Thorax Range
TExc: Torso Excursion
T%V_max_: Time to Peak Velocity
UE: Upper-Extremity
V_max_: Peak Velocity
WMFT: Wolf Motor Function Test
WMFT-FAS: Wolf Motor Function Test Functional Ability Scale
