# Supplementary material for: Can kinematic parameters of 3D reach-to-target movements be used as a proxy for clinical outcome measures in chronic stroke rehabilitation? An exploratory study
Source: J Neuroeng Rehabil. 2020 Aug 8;17:106. doi: 10.1186/s12984-020-00730-1 (PMC7414659; doi:10.1186/s12984-020-00730-1)
Supplement: Supplementary file 1 — Additional file 1: Figure S1A. Variance observed across targets in the kinematic variables of efficiency in the non-paretic and paretic arm. Figure S1B. Variance observed across targets in the kinematic variables of speed and planning in the non-paretic and paretic arm. Figure S1C. Variance observed across targets in the kinematic variables of smoothness in the non-paretic and paretic arm. Figure S1D. Variance observed across targets in the kinematic variables of trunk posture in the non-paretic and paretic arm. Figure S1E. Variance observed across targets in the kinematic variables of arm posture in the non-paretic and paretic arm. [file 12984_2020_730_MOESM1_ESM.pptx]

## Slide 1
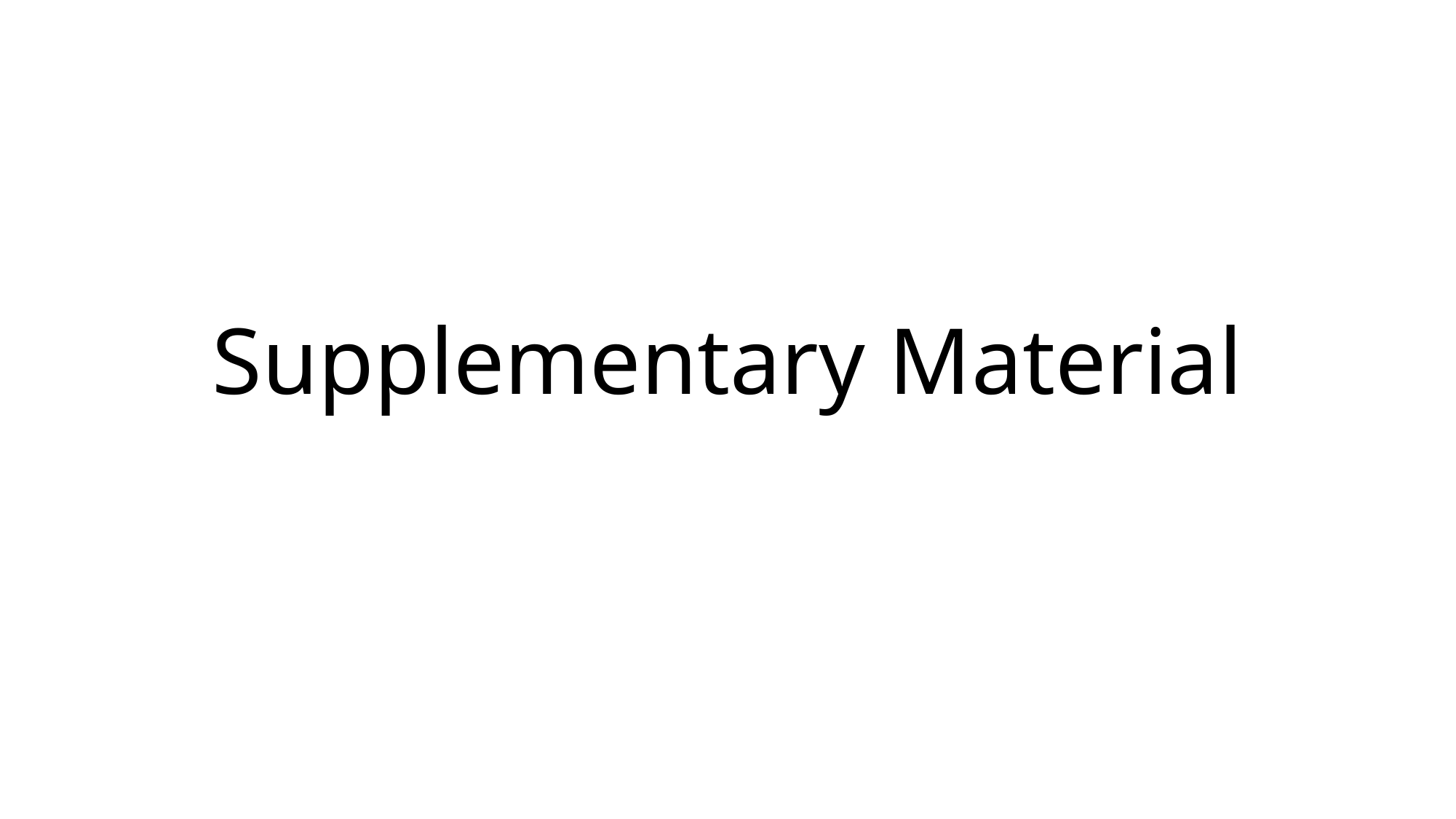

# Supplementary Material

## Slide 2
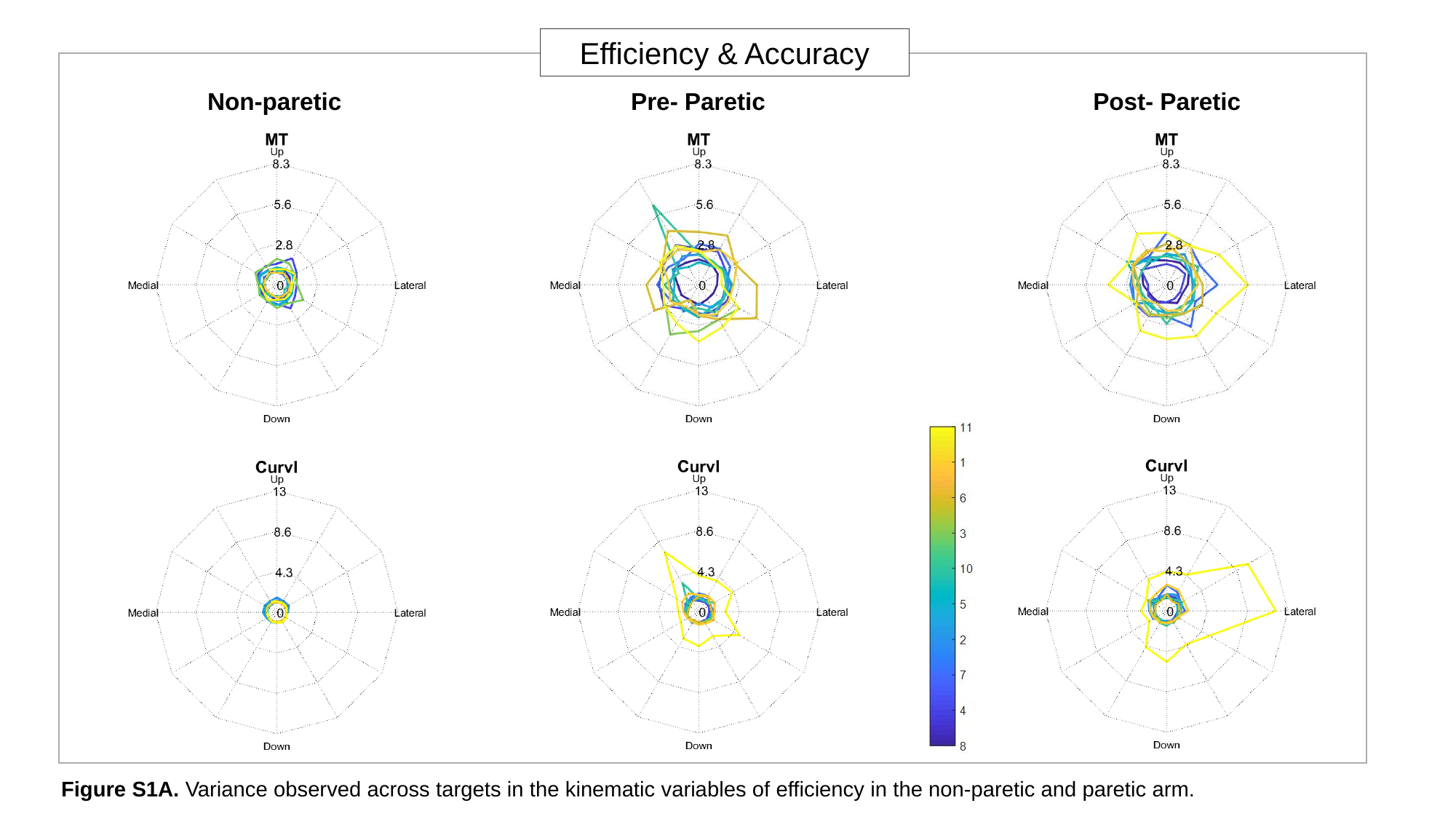

Efficiency & Accuracy
Non-paretic
Pre- Paretic
Post- Paretic
Figure S1A. Variance observed across targets in the kinematic variables of efficiency in the non-paretic and paretic arm.

## Slide 3
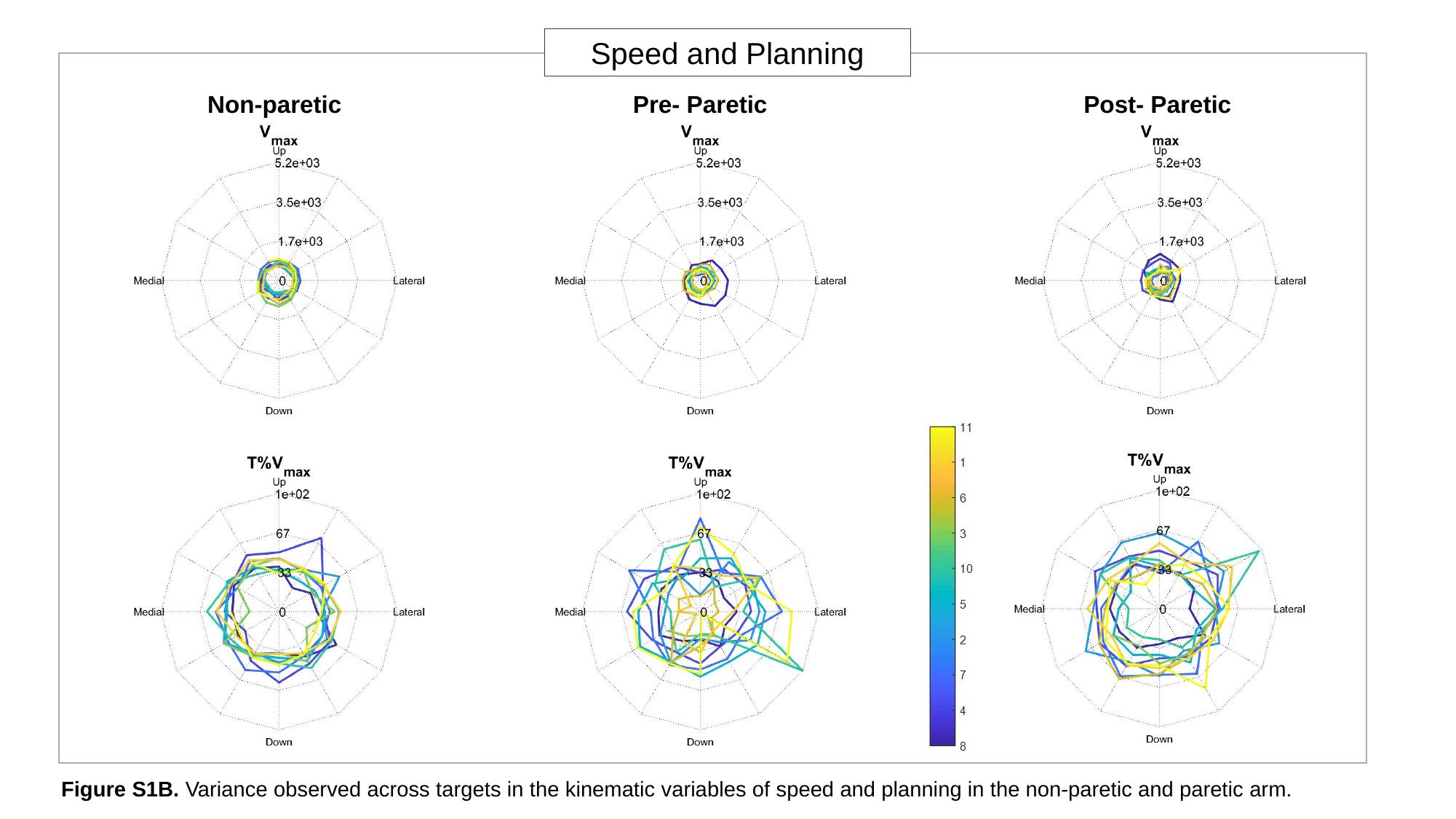

Speed and Planning
Non-paretic
Pre- Paretic
Post- Paretic
Figure S1B. Variance observed across targets in the kinematic variables of speed and planning in the non-paretic and paretic arm.

## Slide 4
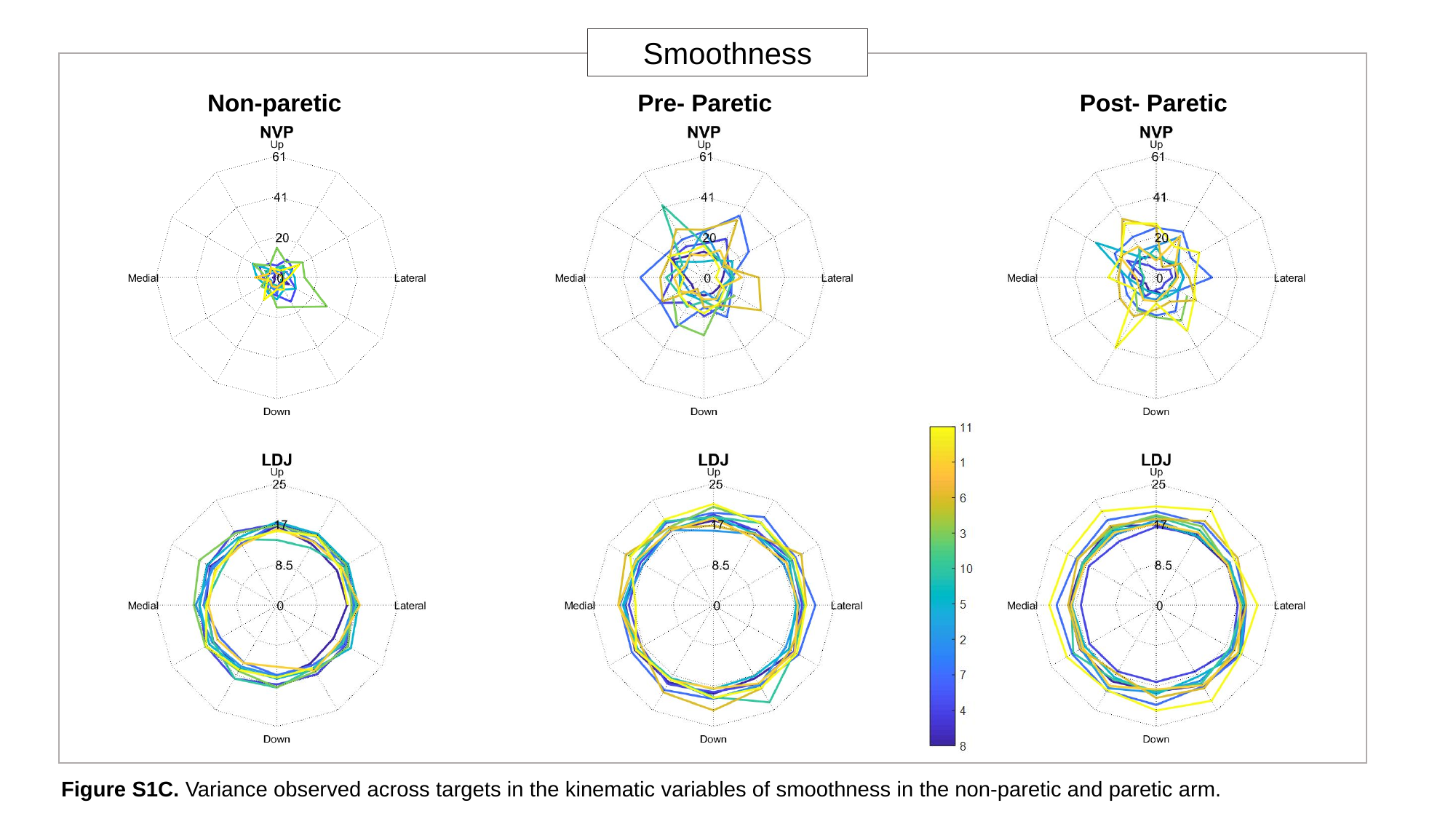

Smoothness
Non-paretic
Pre- Paretic
Post- Paretic
Figure S1C. Variance observed across targets in the kinematic variables of smoothness in the non-paretic and paretic arm.

## Slide 5
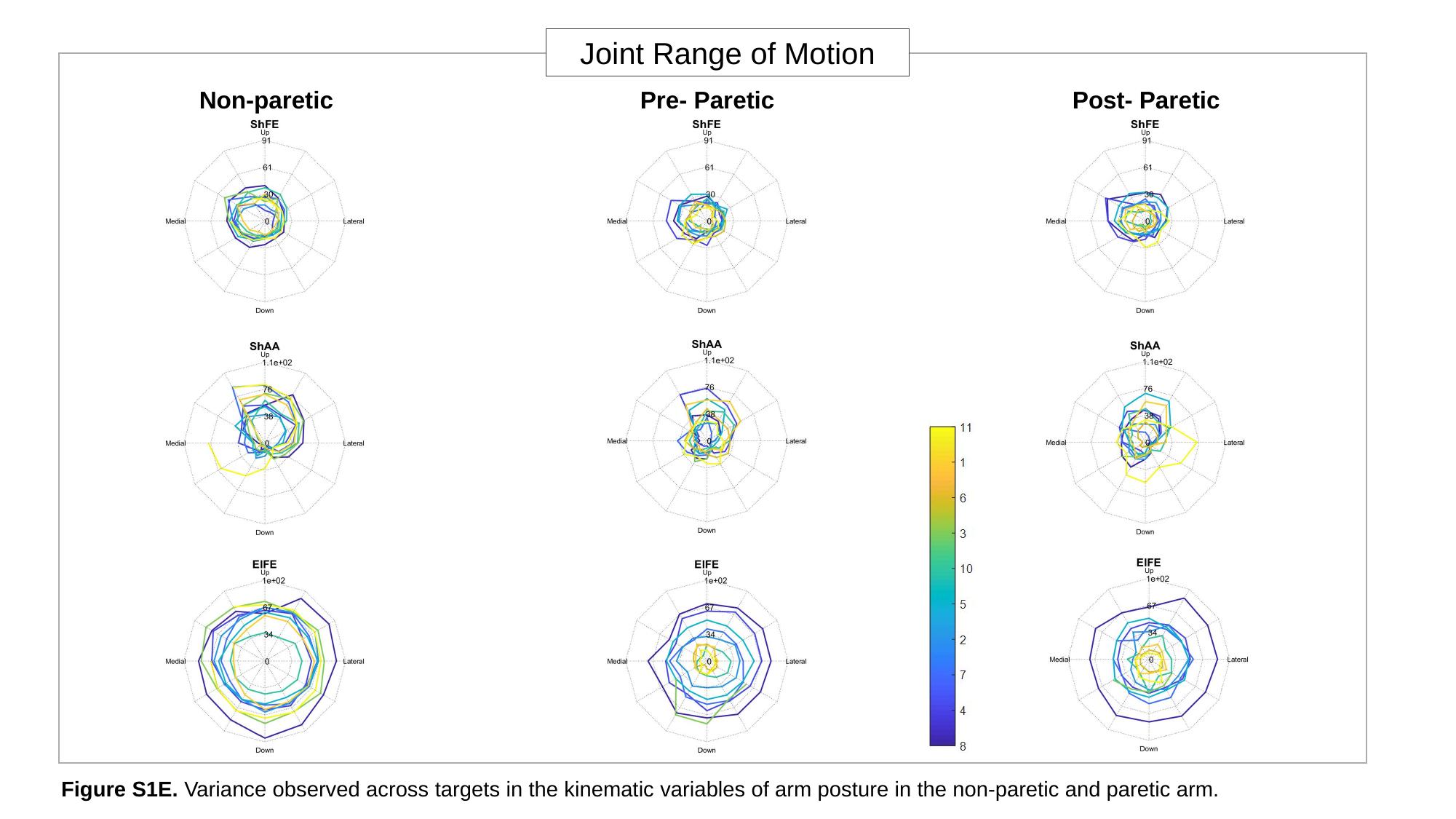

Joint Range of Motion
Non-paretic
Pre- Paretic
Post- Paretic
Figure S1E. Variance observed across targets in the kinematic variables of arm posture in the non-paretic and paretic arm.

## Slide 6
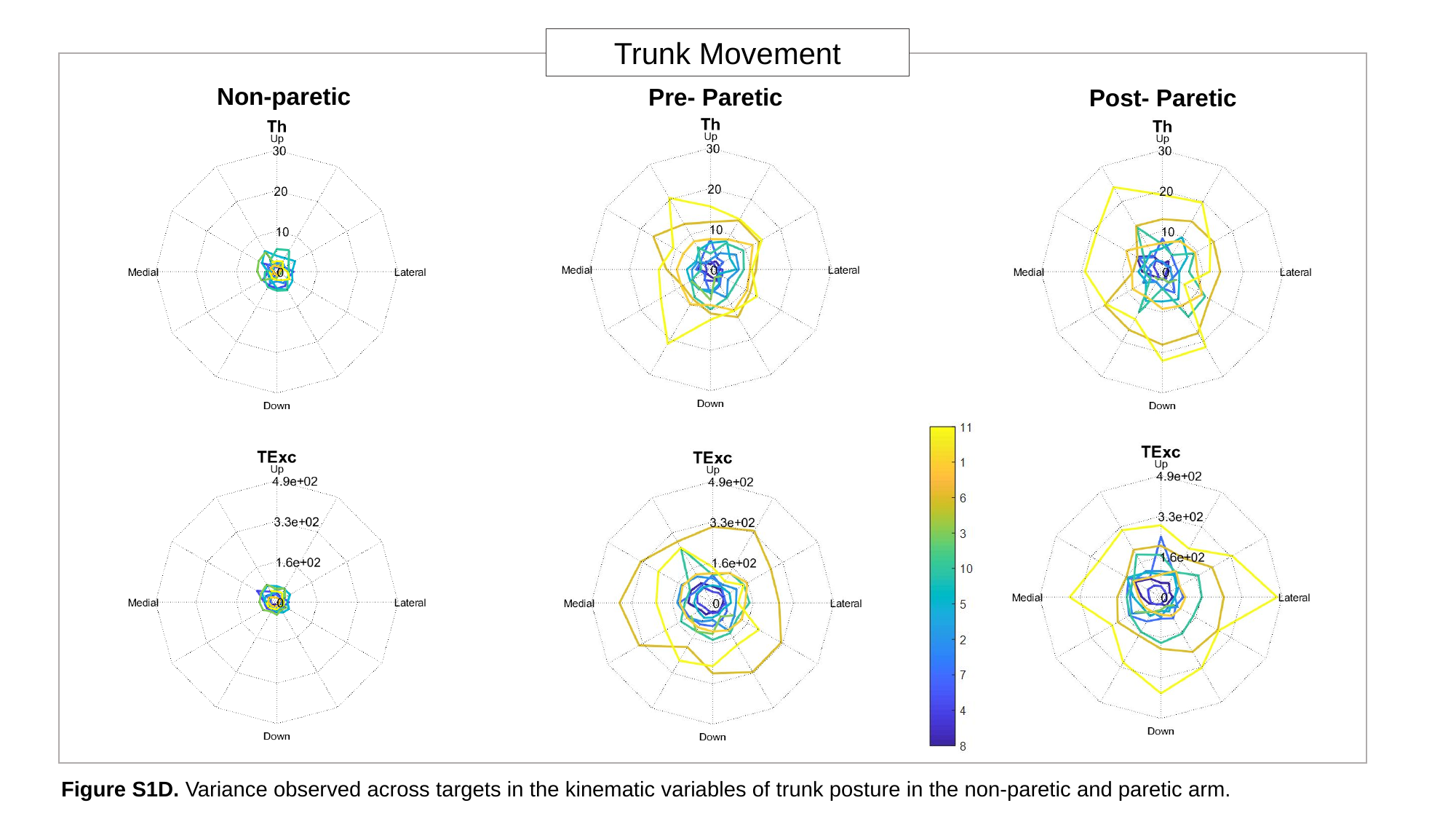

Trunk Movement
Non-paretic
Pre- Paretic
Post- Paretic
Figure S1D. Variance observed across targets in the kinematic variables of trunk posture in the non-paretic and paretic arm.
